# Cambogin exerts anti-proliferative and pro-apoptotic effects on breast adenocarcinoma through the induction of NADPH oxidase 1 and the alteration of mitochondrial morphology and dynamics

**DOI:** 10.18632/oncotarget.10585

**Published:** 2016-07-13

**Authors:** Kaikai Shen, Fangfang Lu, Jianling Xie, Minfeng Wu, Bo Cai, Yurong Liu, Hong Zhang, Hongsheng Tan, Yingyi Pan, Hongxi Xu

**Affiliations:** ^1^ School of Pharmacy, Shanghai University of Traditional Chinese Medicine, Shanghai 201203, China; ^2^ Institute of Arthritis Research, Shanghai Academy of Chinese Medical Sciences, Guanghua Integrative Medicine Hospital/Shanghai University of T.C.M, Shanghai 201203, China; ^3^ Engineering Research Center of Shanghai Colleges for TCM New Drug Discovery, Shanghai 201203, China; ^4^ Nutrition & Metabolism, South Australian Health & Medical Research Institute, North Terrace, Adelaide SA5000, Australia; ^5^ Centre for Biological Sciences, Life Sciences Building, University of Southampton, Southampton, SO17 1BJ, UK

**Keywords:** breast cancer, cambogin, reactive oxygen species, NADPH oxidase 1, thioredoxin-1/ASK1 complex

## Abstract

Cambogin, a bioactive polycyclic polyprenylated acylphoroglucinol (PPAP) derived from the *Garcinia* genus, possesses proapoptotic effect in medulloblastoma and breast cancer cells. We have previously demonstrated that the proapoptotic effect of cambogin is driven by the production of reactive oxygen species (ROS). Here we have shown that the inhibitory effect of cambogin on cell proliferation is associated with the loss of mitochondrial transmembrane potential (Δ*Ψ*_m_) and mitochondrial fragmentation. Cambogin also promotes the mutual complex formation of the membrane-bound subunit p22^phox^ of NADPH oxidase 1 (NOX1), as well as the phosphorylation of the cytosolic subunit p47^phox^, subsequently enhancing membrane-bound NOX1 activity, which leads to increases in intracellular and mitochondrial levels of O_2_^.-^ and H_2_O_2_. Pharmacological inhibition of NOX1 using apocynin (pan-NOX inhibitor), ML171 (NOX1 inhibitor) or siRNA against NOX1 prevents the increases in O_2_^.-^ and H_2_O_2_ levels and the anti-proliferative effect of cambogin. Antioxidants, including SOD (superoxide dismutase), CAT (catalase) and EUK-8, are also able to restore cell viability in the presence of cambogin. Besides, cambogin increases the dissociation of thioredoxin-1 (Trx1) from ASK1, switching the inactive form of ASK1 to the active kinase, subsequently leads to the phosphorylation of JNK/SAPK, which is abolished upon ML171 treatment. The proapoptotic effect of cambogin in breast cancer cells is also aggravated upon knocking down Trx1 in MCF-7 cells. Taken in conjunction, these data indicate that the anti-proliferative and pro-apoptotic effect of cambogin is mediated via inducing NOX1-dependent ROS production and the dissociation of ASK1 and Trx1.

## INTRODUCTION

Breast cancer is the most common malignancy in women worldwide despite early diagnosis and aggressive therapeutic methods [1]. In light of the recognition that alcohol consumption, obesity, lack of physical exercise, hormone replacement therapy during menopause and ionizing radiation are the main risk factors for breast cancer, it is envisaged that the incidence of breast cancer will remain high and the existing treatments unfortunately have only limited efficacy in slowing down clinical decline [2].

Reactive oxygen species (ROS), such as hydrogen peroxide (H_2_O_2_) and the highly reactive hydroxyl radical, are recognized as a second messenger in a variety of cell receptor signal transduction pathways [3]. ROS exerts a pivotal role in a number of cellular processes associated with tumor progression, including survival, proliferation, invasion, angiogenesis and metastasis [4-6]. Most of the currently available anti-cancerous chemotherapeutic, photodynamic and radiotherapeutic agents are selectively toxic to tumor cells by augmenting oxidative stress, resulting in sustained cell-cycle inhibition, cell death induction, and senescence, in the present, this likely represents one of the best opportunities for cancer therapeutics [7-9]. Several intracellular sources can contribute to the production of ROS, such as cyclooxygenase, cytochrome P450, lipoxygenases, mitochondrial electron transport, xanthine oxidase and NADPH oxidase (NOX) [10, 11]. In non-phagocytic cells, the NOX enzymes are the key component of the so-called “redox signaling system” that regulate many cellular responses by modulating intracellular ROS content [12]. NOX is a superoxide-producing enzyme system consisting of membrane-bound subunits, including gp91^phox^ homologues (NOX1-5, Duox1 and Duox2) and p22^phox^; cytosolic subunits (p47^phox^, p67^phox^, and p40^phox^), and the small GPTase Rac [13-15]. Upon activation, the transmembrane subunit p22^phox^ first forms a mutually stabilized complex with gp91^phox^ homologues, followed by the phosphorylation of the cytosolic subunit p47^phox^ and the entire cytosolic complex interacts with the small GTPase Rac and translocate to the plasma membrane, where all components assemble to form the active complex [13, 14, 16, 17]. This system is considered to be the predominant source of ROS accumulation and oxidative stress in response to oncogenic signals as well as DNA-damaging agents in cancer cells.

On the other hand, Garcinia species are tropical evergreen trees and shrubs that are widely distributed in Southeastern Asia and used in folk medicine to promote detoxification in the treatment of inflammation and wounds [18]. Polycyclic polyprenylated acylphloroglucinols (PPAPs), one of the main bioactive components, are known to possess anticancer activities, including the induction of apoptosis, the inhibition of proliferation, and the prevention of cancer metastasis and tumor angiogenesis [19-21]. One of the mechanisms by which PPAPs exert anti-tumor effect is via elevating the levels of ROS [21, 22]. Cambogin is a bioactive PPAP isolated from the branches of *Garcinia esculenta* [23]. Previously we have shown that cambogin induces breast adenocarcinoma cell apoptosis via ROS-mediated increases in Bax/Bcl-2 ratio and the nuclear import of apoptosis inducing factor (AIF), in parallel with the stimulation of ASK1-MKK4/MKK7-JNK/SAPK signaling cascade [21]. However, the underlying mechanism of how cambogin promotes ROS formation remains elusive. In the present study, we identified the NOX system as a crucial regulator of ROS-dependent cell apoptosis in response to cambogin in breast adenocarcinoma.

## RESULTS

### The reduction in breast cancer cell viability in response to cambogin is associated with alterations in mitochondria morphology and dynamics

**Figure 1:**
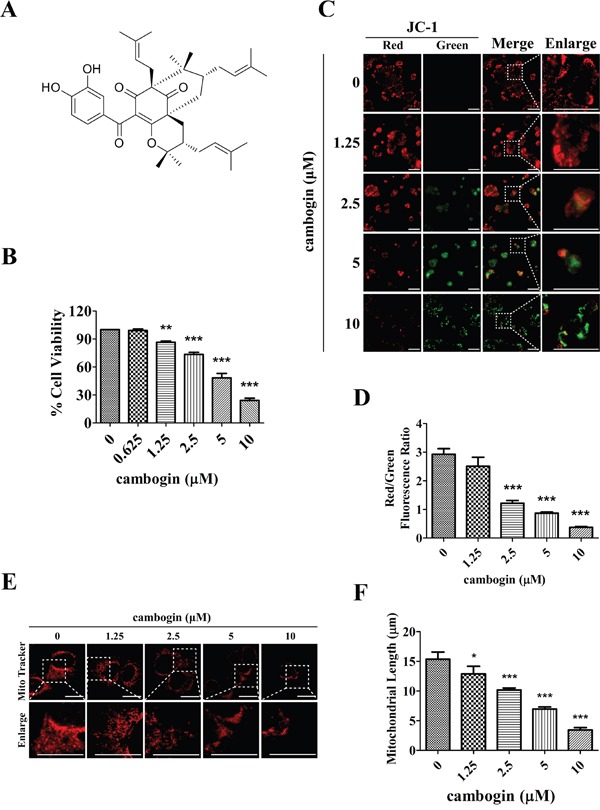
Cambogin inhibits cell proliferation and modulates mitochondrial network in MCF-7 cells **A.** Chemical structure of cambogin. **B.** MCF-7 cells were treated with cambogin (0-10 μM) for 24 h. Cell viability was measured by MTT assay. **C, D.** MCF-7 cells were exposed to cambogin (0-10 μM) for 4 h. The percentage of cells with a reduction in Δ*Ψ*m was measured using the fluorescent dye JC-1 (10 μM). After incubation, stained cells were observed under an inverted fluorescent microscope (C) and were measured by microplate fluorescence reader (D). JC-1 dye changes color as the membrane potential increases. At higher membrane potentials, JC-1 forms aggregates, which changes the fluorescence emission color from green to red. Scale bar=100 μm. **E.** MCF-7 cells were treated with cambogin (0-10 μM) for 24 h. After treatment, the cells were washed, stained with MitoTracker Red for 1 h, washed again, and analyzed for mitochondrial network under confocal microscopy (×1000). Scale bar=20 μm. **F.** Statistical analyses of the average mitochondrial length for experiment E. Data are shown as means±SEM; ^*^P<0.05, ^**^P<0.01, ^***^P<0.001 compared with control. *n*=3.

We have reported previously [21] that cambogin (chemical structure shown in Figure 1A) strongly inhibits cell proliferation in several breast cancer cell lines, including MCF-7 (ER^+^PR^+^HER2^−^), SK-BR-3 (ER^−^PR^−^HER2^+^) and MDA-MB-468 (ER^−^PR^−^HER2^−^, also known as TNBC (triple negative breast cancer)). As shown in Figure 1B, cambogin (0-10 μM) treatment led to a reduction in MCF-7 cell viability in a dose-dependent manner, which can be achieved with a concentration as low as 1.25 μM, whereas at 10 μM it produced the maximal inhibition. The IC_50_ value of cambogin was 4.91 μM for MCF-7 cells. Therefore, we chose to use 1.25 μM, 2.5 μM, 5 μM and 10 μM final concentrations of cambogin treatment, as a weak, low, medium and strong, respectively, inducer of apoptosis throughout the present study.

Depolarization of the mitochondrial transmembrane potential (Δ*Ψ*_m_) has been suggested to be central to the intrinsic apoptotic pathway [24], it is characterized by the permeabilization of the mitochondrial outer membrane, which occurs in response to various stress stimuli and is regulated by the release of a number of apoptogenic factors including members of the Bcl-2 family [25]. Since cambogin has been demonstrated to cause an increase in the pro-apoptotic Bax/anti-apoptotic Bcl-2 ratio in MCF-7 cells [21], we asked whether cambogin affected Δ*Ψ*_m_. We measured Δ*Ψ*_m_ by using the fluorescent dye JC-1. A reduction in Δ*Ψ*_m_ is associated with a decrease in the red/green fluorescent ratio. Breast cancer cells exposed to concentrations of cambogin ranging 1.25 to 10 μM exhibited a dissipation of Δ*Ψ*_m_ in a concentration-dependent manner, as demonstrated by a decrease in red/green ratio in MCF-7 (Figure 1C and 1D), MDA-MB-468 (Supplementary Figure 1A and 1B) and SK-BR-3 cells (Supplementary Figure 1C and 1D). Since Δ*Ψ*_m_ dissipation is often associated with the permeabilization of the outer mitochondrial membrane and oxidative stress results in mitochondrial network abnormalities, we investigated whether cambogin affected mitochondrial network in breast cancer cells. In control cells, mitochondria mainly exhibited a tubular length of 15.4±4.6 μm, a hallmark of well-balanced fission and fusion (Figure 1E and 1F). Cambogin treated cells showed multiple mitochondrial network abnormalities in a dose-dependent manner in MCF-7 cells. For instance, 24 h of treatment with low concentrations of cambogin (1.25 μM) resulted in a modest mitochondrial truncation, as evidenced by a shortened mitochondria average length (12.8±4.0 μm) (Figure 1E and 1F). Substantial mitochondrial fragmentation occurred with 10 μM cambogin treatment, resulting in extremely short mitochondria (average length 3.0±1.5 μm) (Figure 1E and 1F). The majority of the mitochondria became punctate and clustered. Likewise, cambogin induced the alteration of mitochondrial morphology and dynamics in MDA-MB-468 (Supplementary Figure 2A and 2B) and SK-BR-3 cells (Supplementary Figure 2C and 2D). These results suggest that cambogin treatment induces mitochondrial fragmentation and clustering, and subsequently leads to a reduction in Δ*Ψ*_m_, which are associated with apoptosis in MCF-7 cells.

### Cambogin increases intracellular and mitochondrial levels of H_2_O_2_ and hence ROS production

**Figure 2:**
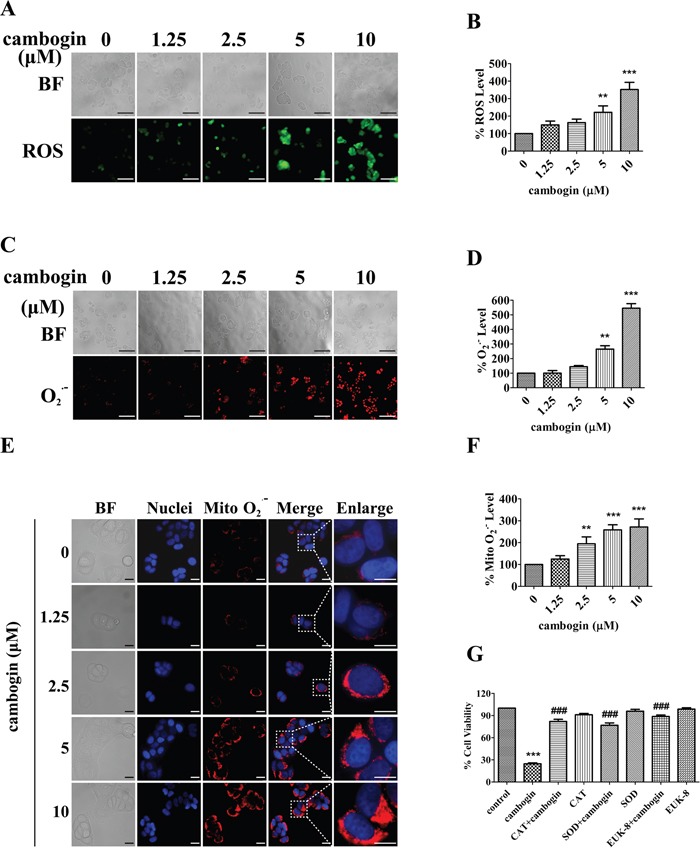
Cambogin induces the generation of ROS in MCF-7 cells **A, B.** MCF-7 cells were treated with cambogin (0-10 μM) for 2 h. The production of H_2_O_2_ was measured using the fluorescent dye DCFH-DA (20 μM). After incubation, stained cells were observed under an inverted fluorescent microscope (A) and the fluorescence intensity units (FIU) were measured at 488 nm (excitation wavelength) and 525 nm (emission wavelength) (B). Scale bar=100 μm. **C, D.** MCF-7 cells were treated with cambogin (0-10 μM) for 2 h. The production of O_2_^.-^ was measured using the fluorescent dye DHE (50 μM). After incubation, stained cells were observed under an inverted fluorescent microscope (C) and FIU was measured at 530 nm (excitation wavelength) and 610 nm (emission wavelength) (D). Scale bar=100 μm. **E, F.** MCF-7 cells were treated with cambogin (0-10 μM) for 2 h. The production of O_2_^.-^ was measured using the fluorescent dye MitoSOX™ (5 μM) and Hoechst 33342 (10 μg/ml). After incubation, stained cells were observed under an inverted fluorescent microscope (E) and FIU was measured at 510 nm (excitation wavelength) and 580 nm (emission wavelength) (F). Scale bar=20 μm. **G.** MCF-7 cells were treated with cambogin for 24 h after pretreatment with CAT (1000 U/ml), SOD (100 U/ml), and EUK-8 (50 μM) for 2 h. Cell viability was measured by MTT assay. Data are shown as means±SEM; ^**^P<0.01, ^***^P<0.001 compared with control; ^###^*P*<0.001 compared with cambogin. *n*=3.

The generation of ROS plays a critical role in the proapoptotic activities of various anticancer agents [26-28]. In MCF-7 cells, cambogin caused a concentration-dependent increase in ROS production, with the minimal effective concentration of 5 μM (Figure 2A and 2B). A dose-dependent increase in intracellular O_2_^.-^ levels was detected in MCF-7 cells following incubation with cambogin (Figure 2C and 2D). Cells were treated with increasing doses of cambogin, and were then stained with the mitochondria-targeting dye MitoSOX™, which serves as a fluoroprobe for selective detection of superoxide in the mitochondria. Cambogin treatment substantially increased mitochondrial O_2_^.-^ levels in a dose-dependent manner in MCF-7 cells (Figure 2E and 2F), and this is accompanied by an increase in the production of H_2_O_2_ but not nitric oxide (NO) (Supplementary Figure 2E and 2F). It can be inferred from the fact that the enhancement of ROS generation is attributed to the production of mitochondrial O_2_^.-^ as well as intracellular O_2_^.-^. Antioxidants, including SOD (O_2_^.-^ scavenger), CAT (H_2_O_2_ scavenger) and EUK-8 (synthetic catalytic superoxide and hydrogen peroxide scavenger), significantly prevented the inhibitory effect of cambogin on cell proliferation at 24 h by MTT assay (Figure 2G) and by SYBR Green assay (Supplementary Figure 3A).

### The activation of NOX is crucial for cambogin-mediated ROS production

**Figure 3:**
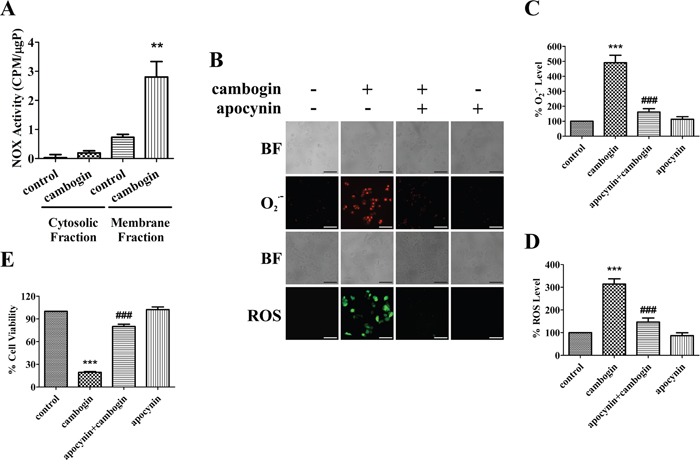
The activation of NOX in response to cambogin is dependent on the generation of ROS **A.** MCF-7 cells were incubated in the absence or presence of cambogin (10 μM) for 2 h. NOX activity was measured and the enzyme activity is expressed as relative light units (RLU). **B.** MCF-7 cells were incubated in the absence or presence of cambogin (10 μM) for 2 h after pretreatment with apocynin (500 μM) for 2 h. The production of O_2_^.-^ and H_2_O_2_ was measured by DHE (for O_2_^.-^) and DCFH-DA (for H_2_O_2_) staining. After incubation, stained cells were observed under an inverted fluorescent microscope. Scale bar=100 μm. **C.** The FIU of O_2_^.-^ was measured at 530 nm (excitation wavelength) and 610 nm (emission wavelength). **D.** The FIU of H_2_O_2_ was measured at 488 nm (excitation wavelength) and 525 nm (emission wavelength). **E.** MCF-7 cells were treated with cambogin for 24 h after pretreatment with apocynin (500 μM) for 2 h. Cell viability was measured by MTT assay. Data are shown as means±SEM; ^**^P<0.01, ^***^P<0.001 compared with control; ^###^*P*<0.001 compared with cambogin. *n*=3.

The superoxide anion radical generating enzymes NOX are dedicated to the specific and deliberate production of free radicals [13]. Cambogin evoked a substantial increase in NOX activity in the membrane fractions of treated cells in comparison with the untreated ones in MCF-7 (Figure 3A), MDA-MB-468 (Supplementary Figure 4A) and SK-BR-3 (Supplementary Figure 4B). In contrast, NOX activity was merely detectable in both control and cambogin-treated cells in the cytosolic fractions, implying that cambogin enhances the activity of membrane-bound NOX in breast cancer cells (Figure 3A and Supplementary Figure 4). Besides, pan-NOX inhibitor apocynin prevented cambogin-induced production of O_2_^.-^ and H_2_O_2_ (Figure 3B-3D) and the pro-apoptotic effect of cambogin was also abolished by apocynin (Figure 3E and Supplementary Figure 3B). These results strongly suggest that the NOX system is crucial for the generation of ROS in response to cambogin.

### Cambogin enhances the interaction between NOX1 and p22^phox^

**Figure 4:**
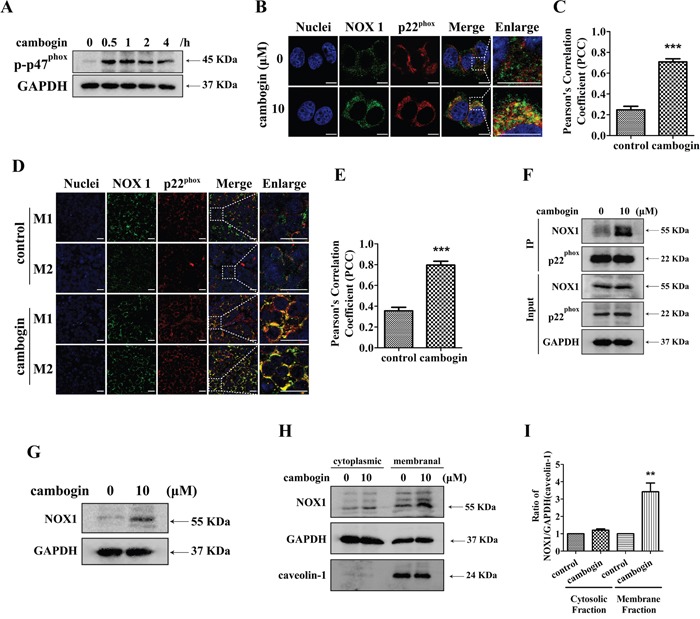
Cambogin enhances the co-localization of NOX1 and p22^phox^ **A.** MCF-7 cells were treated with cambogin (10 μM) for the indicated time periods (0-4 h) and the expression of p-p47^phox^ and GAPDH were analyzed by SDS-PAGE/Western blotting. **B, C.** Immunofluorescent staining for NOX1 (green) and p22^phox^ (red) in the MCF-7 cells after treatment with cambogin (10 μM) for 2 h. The co-localization of NOX1 and p22^phox^ was observed under confocal microscopy (×630) (B) and PCC was applied to statistically quantify the co-localization (C). Scale bar=10 μm. **D, E.** Immunofluorescent staining for NOX1 (green) and p22^phox^ (red) in the tumor section after treatment with cambogin (10 mg/kg) for 35 days. The co-localization of NOX1 and p22^phox^ was observed with confocal microscopy (×630) (D) and PCC was applied as statistic quantification for colocalization (E). Scale bar=20 μm. **F.** MCF-7 cells were incubated in the absence or presence of cambogin (10 μM). p22^phox^ was immunoprecipitated from cell lysates using anti-p22^phox^ antibody. The immunoprecipitates were then run on a SDS-PAGE gel and probed for anti-NOX1 and anti-p22^phox^. **G.** MCF-7 cells were treated with cambogin for 2 h. The expression of NOX1 and GAPDH was determined by SDS-PAGE/Western blotting. **H.** MCF-7 cells were treated with cambogin (10 μM) for 2 h. The expression of NOX1 in the cytoplasmic and membranal fractions was determined by SDS-PAGE/Western blotting. **I.** Data are expressed as ratios to GAPDH (cytoplasmic fractions) or caveolin-1 (membranal fractions). Data are shown as means±SEM; ^**^P<0.01, ^***^P<0.001 compared with control. *n*=3.

The activation of NOX initiates when the membrane-bound subunit p22^phox^ forms a mutually stabilizing complex with the NOX isoforms, followed by the phosphorylation of the cytosolic subunit p47^phox^, and subsequently the entire cytosolic complex associates with the small GTPase Rac which translocates it to the plasma membrane, where all components assemble to form the active complex [16]. In MCF-7 cells, exposure to cambogin stimulated the tyrosine phosphorylation of p47^phox^ within 30 min of incubation, and this phosphorylation event persisted for up to at least 4 h (Figure 4A). Cambogin treatment also enhanced the interaction between NOX1 and p22^phox^ (Figure 4B). We used Pearson's correlation coefficient (PCC) to quantitatively determine the rates of co-localization between NOX1 and p22^phox^ (PCC value close to zero reflects poor co-colocalization). In consistent with results from the confocal microscopy, PCC values of cambogin-treated cells were significantly higher than that of the untreated ones (Figure 4C). Previously, we have demonstrated that cambogin is able to suppress breast adenocarcinoma tumor growth without any apparent toxicity *in vivo*, with a decrease of 73% of tumor size at the dose of 10 mg/kg [21]. We also isolated the tumor tissue (MCF-7 xenograft) from animals treated with cambogin (10 mg/kg) for 35 days, consistent with the *in vitro* data (Figure 4B and 4C), cambogin indeed enhanced the interaction of NOX1 and p22^phox^ *in vivo* (Figure 4D and 4E). Likewise, cambogin induced an increase in the binding of NOX1 to p22^phox^ after 2 h of cambogin treatment in MCF-7 cells (Figure 4F). We observed an increase in the expression of NOX1 in the membrane fraction as well as total NOX1 when cells were treated with cambogin (Figure 4G and 4H). However, there was little change in the expression of cytosolic NOX1 (Figure 4H and 4I), suggesting that cambogin treatment alters the assembling and localization of NOX1.

**Figure 5:**
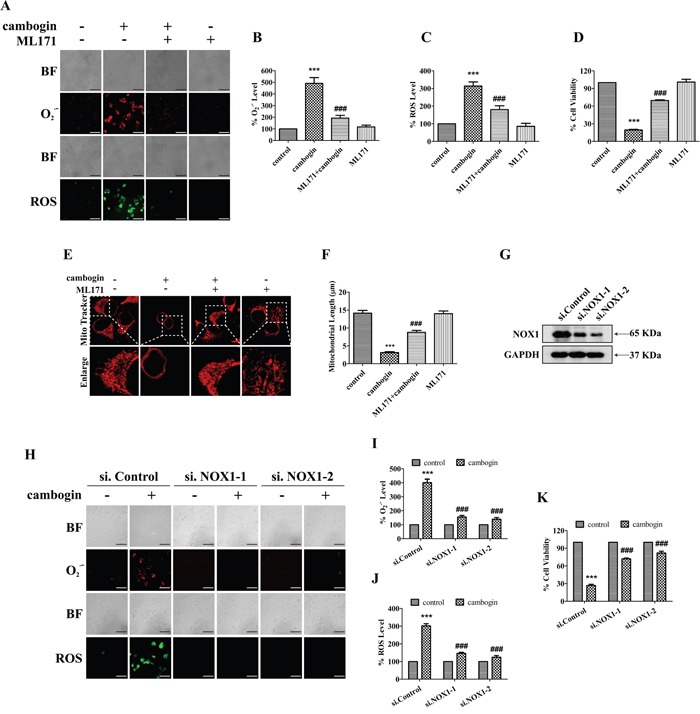
NOX1 is required for cambogin-stimulated generation of ROS **A.** MCF-7 cells were incubated in the absence or presence of cambogin (10 μM) for 2 h after pretreatment with ML171 (20 μM) for 2 h. The production of O_2_^.-^ and H_2_O_2_ were measured by DHE (for O_2_^.-^) and DCFH-DA (for H_2_O_2_) staining. After incubation, stained cells were observed under an inverted fluorescent microscope. Scale bar=100 μm. **B.** The FIU of O_2_^.-^ was measured at 530 nm (excitation wavelength) and 610 nm (emission wavelength). **C.** The FIU of H_2_O_2_ was measured at 488 nm (excitation wavelength) and 525 nm (emission wavelength). **D.** MCF-7 cells were treated with cambogin for 24 h after pretreatment with ML171 (20 μM) for 2 h. Cell viability was measured by MTT assay. **E.** MCF-7 cells were treated with cambogin for 24 h after pre-treatment with ML171 (20 μM) for 2 h. The cells were then washed with HBSS by three times, stained with MitoTracker Red for 1 h, washed, and analyzed for mitochondrial network under confocal microscopy (×1000). Scale bar=20 μm. **F.** Statistical analyses of the average mitochondrial length for E. **G.** MCF-7 cells were transiently transfected with two independent NOX1 siRNAs (si.NOX1-1 and si.NOX1-2) or control scrambled siRNA (si.Control) for 48 h, NOX1 protein expression levels were determined by SDS-PAGE/Western blotting. GAPDH was served as a loading control. **H.** The production of O_2_^.-^ and H_2_O_2_ was measured by DHE (for O_2_^.-^) and DCFH-DA (for H_2_O_2_) staining in cambogin-inhibited, NOX1-knocked down (as in G) MCF-7 cells. Scale bar=100 μm. **I.** The FIU of O_2_^.-^ was measured at 530 nm (excitation wavelength) and 610 nm (emission wavelength). **J.** The FIU of H_2_O_2_ was measured at 488 nm (excitation wavelength) and 525 nm (emission wavelength). **K.** Cell viability was determined in cambogin-inhibited, NOX1-knocked down MCF-7 cells for 24 h. Data are shown as means±SEM; ^***^P<0.001 compared with control; ^###^*P*<0.001 compared with cambogin-treated cells. *n*=3.

To determine the role of NOX1 in cambogin-induced cell apoptotic responses, we used a specific NOX1 inhibitor ML171 [29] as well as NOX1 siRNAs to either inhibit NOX1 activation or knock down NOX1, respectively. ML171 prevented the increases in O_2_^.-^ and H_2_O_2_ production (Figure 5A-5C), cell proliferation inhibition (Figure 5D and Supplementary Figure 3C) and mitochondrial network abnormalities (Figure 5E and 5F) induced by cambogin. On the other hand, NOX1 siRNAs effectively reduced endogenous NOX1 expression (Figure 5G). Cambogin-induced increases in O_2_^.-^ and H_2_O_2_ formation (Figure 5H-5J), as well as pro-apoptotic response (Figure 5K and Supplementary Figure 3D) were substantially attenuated by NOX1 siRNAs. These results suggest that NOX1 is responsible for cambogin-mediated ROS production and mitochondrial network abnormalities.

### Cambogin causes the dissociation of thioredoxin-1 (Trx1) from ASK1

**Figure 6:**
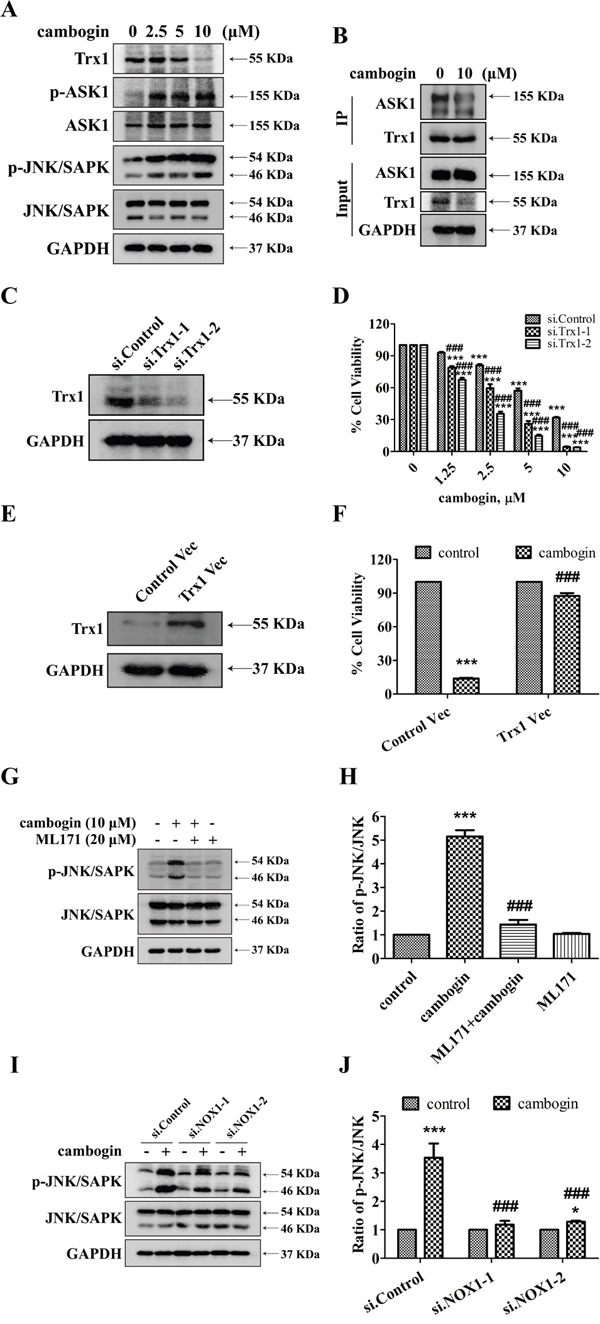
Cambogin leads to the dissociation of Trx1/ASK1 complexes and induces the phosphorylation of ASK1 **A.** Representative western blots for Trx1, phospho-ASK1, ASK1, phospho-JNK/SAPK, JNK/SAPK and GAPDH in MCF-7 cells stimulated with cambogin (0-10 μM) for 24 h. **B.** MCF-7 cells were incubated in the absence or presence of cambogin (10 μM). Trx1 was immunoprecipitated from cell lysates using anti-Trx1 antibodies. The immunoprecipitates were run on a SDS-PAGE gel, the upper half of the blot was probed with anti-ASK1 (top), and the bottom half with anti-Trx1. **C.** MCF-7 cells were transiently transfected with two independent Trx1 siRNA (si.Trx1-1 and si.Trx1-2) or control scrambled siRNA (si.Control) for 48 h, levels of Trx1 were determined by SDS-PAGE/Western blotting. GAPDH was served as a loading control. **D.** MCF-7 cells were transfected with si.Control, si.Trx1-1 or si.Trx1-2 for 24 h, before treated with cambogin for another 24 h. Cell viability was determined by MTT assay. **E.** MCF-7 cells were transiently transfected with Trx1 (Trx1 Vec) or empty vector (Control Vec) for 48 h, levels of Trx1 were determined by SDS-PAGE/Western blotting. GAPDH was served as a loading control. **F.** MCF-7 cells were transfected with Control Vec or Trx1 Vec for 24 h, before treated with cambogin for another 24 h. Cell viability was determined by MTT assay. **G.** MCF-7 cells were treated with cambogin for 24 h after pretreatment with ML171 (20 μM) for 2 h. The expression of phospho-JNK/SAPK and JNK/SAPK was determined by SDS-PAGE/Western blotting. **H.** The data are expressed as ratios to total JNK/SAPK. **I.** Representative blots for phospho-JNK/SAPK and JNK/SAPK in either control or NOX1-knocked down MCF-7 cells treated with vehicle control or cambogin (10 μM) for 24 h. **J.** The data are expressed as ratios to total JNK/SAPK. Data are shown as means±SEM; ^***^P<0.001 compared with control; ^###^*P*<0.001 compared with cambogin. *n*=3.

We have previously shown that the activation of ASK1/JNK signaling cascade plays an essential role in the anti-proliferative and pro-apoptotic effects of cambogin on breast cancer cells [21]. We applied 2D-gel map analysis combined with tandem mass spectrometry to identify targets that are affected by cambogin treatment in human breast cancer cells. We have identified changes in 53 proteins that are highly related to ROS, and further analysis (by using IPA) has revealed that changes in 12 proteins are highly related to Trx1. It has also been reported that ROS induces apoptosis through the oxidation of ASK1 upstream inhibitor Trx1, and hence releasing ASK1 from its inhibitory binding [30]. In MCF-7 cells, cambogin treatment induced a significant increase in the phosphorylation of ASK1 at Thr845 and JNK/SAPK at Thr183 and Tyr185, concomitant with a decrease in Trx1 expression in a dose-dependent manner (Figure 6A). We investigated the effects of cambogin on the binding of Trx1 to ASK1 as an indicator of the functional redox state of Trx1. As shown in Figure 6B, ASK1 was associated with Trx1 in untreated cells; in contrast, cambogin induced a reduction in the binding of ASK1 to Trx1 after 24 h of treatment. Moreover, the pro-apoptotic effect of cambogin in breast cancer cells was further enhanced in siRNA-mediated Trx1 knocked down MCF-7 cells (Figure 6C and 6D and Supplementary Figure 3E). The proapoptotic effect of cambogin in breast cancer cells was attenuated upon exogenously expressing Trx1 in MCF-7 cells (Figure 6E and 6F and Supplementary Figure 3F). The phosphorylation of JNK/SAPK in response to cambogin was effectively abolished by the treatment with ML171 (Figure 6G and 6H) or the knocking down NOX1 using siRNA (Figure 6I and 6J). Collectively, these results indicate that cambogin induces ROS production, which leads to the dissociation of Trx1 from ASK1, switching the inactive form of ASK1 to the active kinase, and subsequently activates JNK/SAPK, and ultimately causes cancer cell death.

## DISCUSSION

It has been well established that ROS are in principal cytotoxic and mutagenic to the cells, and high levels of ROS lead to cell death or senescence [31]. Tumor cells can be inherently more resistant to oxidative stress than normal cells, and therefore oxidative stress may provide a selective advantage in tumor growth [31]. High ROS production also contributes to the cytotoxicity in response to anticancer drugs such as paclitaxel [28]. Indeed, we have previously demonstrated that the pro-apoptotic effect of cambogin in breast cancer cells is driven by high levels of ROS [21]. However, previous to this study, it was not known how ROS production is elevated in response to cambogin, and, in particular, how ROS production affects the apoptosis-dependent downstream signaling pathways in breast cancer cells.

Conventionally, intracellular ROS are mainly derived from the mitochondria and they are generated via electron leakage from mitochondrial electron transport, resulting in the regulation of oncogenic pathways [32]. Here we have shown that cambogin treatment increases mROS (mitochondrial ROS) accumulation in breast cancer cells. On the other hand, plasma membrane-associated NOX is the main source of the extracellular release of O_2_^.-^. The activation of NOX is induced by the assembly of an active enzyme complex comprised of the catalytic subunits gp91^phox^, p22^phox^ and other components [13-17]. Cambogin is able to enhance membrane-bound NOX activity, leading to increased O_2_^.-^ production, which is subsequently converted to H_2_O_2_. The inhibition of NOX by specific inhibitor apocynin or knocking down NOX using siRNA suppresses cambogin-induced elevation of ROS. Emerging evidence indicates that the production of ROS generated by the NOX enzymes is essential for oncogenesis and tumor progression [33]. Among the NOX family members, NOX1, in particular, has been responsible for elevated ROS levels in 80% of all human breast cancers [34]. The present data show that the generation of ROS is responsible for cambogin-induced interaction of p22^phox^ and NOX1 in breast cancer cell membranes both *in vitro* and *in vivo*. This is evidenced by the fact that both NOX1 inhibitor ML171 or siRNA against NOX1 are able to prevent the elevation of O_2_^.-^ and H_2_O_2_ formation in response to cambogin.

Antioxidant enzymes, such as SOD, are ubiquitously expressed inside the cytoplasm (Cu^2+^/Zn^2+^ SOD), mitochondria (Mn^2+^ SOD) or outside (Cu^2+^/Zn^2+^ SOD) the cell (also known as EC-SOD) [35]. Although EC-SOD is mainly found in the extracellular matrix across all tissues, intracellular EC-SOD can also be detected [36]. The uptake of these antioxidative enzymes is not very well studied, but they did effectively prevent the inhibitory effect of cambogin on breast cancer cell proliferation. Therefore, all our data combined indicate that breast cancer progression can be effectively prevented by targeting the ROS/NOX1-dependent signaling transduction pathway, potentially providing several therapeutic targets for breast cancer treatment.

Cancer cells mainly rely on mitochondrial metabolism as their energy source due to their particular growth characteristics, implying that targeting mitochondria may constitute a relevant strategy for inhibiting tumor cell proliferation or inducing tumor cell apoptosis, especially in breast cancer [37]. Considering the fact that cambogin, a potent ROS-inducer, is able to selectively induce mitochondria abnormalities and apoptosis in breast cancer cells rather than normal cells; it is possible that the induction of changes in mitochondrial metabolism contributes to the pro-apoptotic effect of cambogin in breast cancer cells. An interesting and important concept is “mitochondrial criticality” which in some situations determines the life or death of a cell [38], it includes oscillations in mitochondrial energetics, inducing a state in which the mitochondrial network of cancer cells becomes very sensitive to small perturbations in ROS, resulting in the loss of Δ*Ψ_m_* to the whole cells [39]. Indeed, cambogin induced both mROS accumulation and Δ*Ψ_m_* dissipation in breast cancer cells. Emerging evidence indicates that mitochondrial clustering precedes cytochrome c release and an increase in the ratio of Bax/Bcl-2 during etoposide-, paclitaxel- or curcumin-induced apoptosis in breast cancer cells [40-42]. It is likely that clustering of punctate mitochondria results from mitochondrial swelling, a consequence of mitochondrial dysfunction and integrity collapse. In view of previous studies [21] and present findings, the heavy fragmentation of mitochondria into punctuation and their clustering was specifically associated with cell damage as well as nuclear fragmentation and chromatin condensation, representing as hallmarks of breast cancer cell apoptosis in response to cambogin. A previous study from Lee et al. has shown that mROS is an essential trigger but not enough to promote cell death, which requires the sustained accumulation of ROS upon the subsequent activation of NOX1 [43]. Our findings indicated that the blockage of NOX1 activity attenuated the elevation of ROS production and mitochondrial network abnormalities induced by cambogin, further supporting the notion that NOX1 contributed to cambogin-induced mitochondrial disorders, which ultimately leading to apoptosis. These observations highlight the fact that mitochondria are important constituents of the positive feedback loops leading to mROS-triggered ROS production with subsequent mitochondria dysfunctions. Indeed, mitochondria and NOX1 also play a pivotal role in the progression of the pro-apoptotic effect in many other types of cancer [33, 34, 38]; it can be tempting to speculate that targeting tumor-specific mitochondrial network abnormalities can be further exploited in the future to enhance the induction of tumor-targeting apoptosis.

ASK1, a ubiquitously expressed MAP kinase kinase kinase 5 (MAP3K5), is the central mediator of a prominent pathway that leads to cell apoptosis. When being active, ASK1 exists as a signalosome which can be found binding to another ASK1 molecule (homo-oligomerization). In contrast, in its inactive state, ASK1 is associated with negative regulators such as Trx1 [44], phosphatase 2C epsilon [45] and 14-3-3 [46]. For instance, Trx1 binds to the *N* terminal non catalytic region of ASK1 under inhibitory conditions, upon ROS stimulation, residues Cys32 and Cys35 within Trx1 are oxidized and form an intramolecular disulfide, which leads to the subsequent dissociation of Trx1 from ASK1, facilitating ASK1 activation [47, 48]. Activation of ASK1 in turn results in the phosphorylation of its downstream substrates such as p38 and JNK/SAPK, both of which have been reported to be implicated in the propagation of cell apoptosis [49]. Our previous study [21], together with the data presented here, indicate that cambogin treatment induces the activation of ASK1/JNK, but not that of p38 MAPK, in a time- and concentration-dependent manner. Supporting this view, co-immunoprecipitation studies using anti-Trx1/ASK1 antibodies have also demonstrated that cambogin treatment significantly disturbed the binding between Trx1 and ASK1. Trx1 is essential for cell survival *in vitro* [50], and Trx2 knockout mice exhibit early embryonic lethality [51]. Inhibition of or knocking down Trx1 promotes oxidative stress as well as increases the sensitivity of cells to oxidants and redox-mediated apoptosis [52, 53]. Conversely, cells that overexpress Trx1 are more resistant to oxidant-induced apoptosis [52, 53]. The interaction between Trx1 and ASK1 is highly dependent on the redox status of Trx1 and the oxidative state of Trx is unable to bind ASK1. The present findings provide direct evidence that Trx1 can be oxidized during cambogin treatment. Notably, we observed a reduction in Trx1 protein levels upon cambogin treatment. Although oxidative stress-induced Trx1 degradation has already been reported elsewhere [54, 55], the underlying mechanism remains obscure, thereby how cambogin induces the reduction in Trx1 expression also awaits further study. Of note, the pro-apoptotic effect of cambogin was further aggravated when the cells were transiently transfected with siRNA against Trx1, suggesting that Trx1 inhibitors may enhance the effectiveness of cambogin in breast cancer treatment. Under the experimental conditions presented in this study, ROS is more likely to act as inducers of cancer cell apoptosis.

Although ASK1 can be activated by a number of stress stimuli including ROS in multiple cell types, its role as a downstream target of the NOX enzymes is still obscure. Before this report, we can only find out one study in the literature suggesting an interaction between ASK1 activation and NOX1 in vascular disease [56]. Our findings support the notion that there is a direct link between NOX1 and ASK1 which plays an important role in mediating breast cancer cell viability. We also demonstrated that the activation of ASK1 lies downstream of NOX1-derived ROS production in cambogin-treated breast cancer cells. Notably, the inhibition of NOX1 was sufficient to prevent the dissociation of Trx1 from ASK1 and hence the activation of ASK1/JNK, implying that targeting this pathway is likely to be a promising strategy for the treatment of breast cancer.

In conclusion, our findings indicate that in breast cancer cells, in response to cambogin treatment, the NOX enzyme is activated through the enhancement of p22^phox^ and NOX1 interaction, and ROS derived from NOX stimulation subsequently leads to the dissociation of Trx1 from ASK1, resulting in the activation of ASK1/JNK pathway and the induction of mitochondrial network abnormalities, leading to the inhibition of breast adenocarcinoma cell proliferation and ultimately cell death. This work has a great impact on our understanding of the importance of ROS signaling pathway in breast cancer cell survival, and hence has provided more promising future therapeutic targets in the ongoing battle against breast cancer.

## MATERIALS AND METHODS

### Chemical compound

Cambogin was isolated from the branches of *Garcinia esculenta*. Its structure was determined using ^1^H-NMR and ^13^C-NMR spectral analysis, the achieved purity was more than 98% as determined by high performance liquid chromatography analysis. Cambogin was dissolved in absolute dimethyl sulfoxide (DMSO), and, on the experiment day, was further diluted with culture medium.

### Cell culture and transfection

The human breast cancer cell lines (MCF-7, SK-BR-3 and MDA-MB-468) were purchased in 2013 from the Chinese Academy of Science Committee Type Culture Collection Cell Bank (Shanghai, China). MCF-7 cells were cultured in Dulbecco's Modified Eagle Medium (DMEM, Invitrogen) with 10% (v/v) fetal bovine serum (FBS, Invitrogen), 0.01mg/ml bovine insulin (Sigma) and 1% penicillin/streptomycin (Invitrogen) at 37°C in a humidified atmosphere with 5% CO_2_. SK-BR-3 cells were cultured in Dulbecco's Modified Eagle Medium (DMEM, Invitrogen) with 10% (v/v) fetal bovine serum (FBS, Invitrogen) and 1% penicillin/streptomycin (Invitrogen) at 37°C in a humidified atmosphere with 5% CO_2_. MDA-MB-468 cells were cultured in L15 medium (Invitrogen) with 10% (v/v) FBS and 1% penicillin/streptomycin at 37°C in a humidified atmosphere with 0% CO_2_. Authenticity of these cell lines was performed using the standard short tandem repeat (STR) DNA typing methodology, by the Chinese Academy of Science Committee Type Culture Collection Cell Bank before purchase. No authentication of these cell lines was done by the authors. Cells were expanded and frozen in multiple vials after the 3^rd^ generation and passaged in culture for no more than 4 months after being thawed from the stocks. Culture medium was replaced every 2-3 days. Confluent cells were split at 1:3 ratios for maintenance. Cells were routinely tested for mycoplasma contamination.

In gene silencing experiments, MCF-7 cells were transfected with either a scramble siRNA or siRNAs against NOX1 or Trx1 using the lipofectamine RNA/MAX kit (Invitrogen) according to the manufacturer's instructions. siRNAs against Trx1-1: 5′-CCA CCA UUA AUG AAU UAG UTT-3′ (sense) and 5′-ACU AAU UCA UUA AUG GUG GTT-3′(antisense); Trx1-2: 5′-CUG CAG GUG AUA AAC UUG UTT-3′ (sense) and 5′-ACA AGU UUA UCA CCU GCA GTT-3′ (antisense); NOX1-1: 5′-GCC GAC AAA UAC UAC UAC ATT-3′ (sense) and 5′-UGU AGU AGU AUU UGU CGG CTT-3′ (antisense); NOX1-2: 5′-GAU CCU AGA AAG GUU CAA UTT-3′ (sense) and 5′-AUU GAA CCU UUC UAG GAU CTT-3′ (antisense); scramble control, 5′-UUC UCC GAA CGU GUC ACG UTT-3′ (sense) and 5′-ACG UGA CAC GUU CGG AGA ATT-3′ (antisense). Plasmid transfection was performed using X-tremeGENE HP DNA Transfection (Roche, Penzberg, Germany) according to the manufacturer's protocol.

### Cell viability assay

Where indicated, cells were pretreated with 1000 U/ml catalase (CAT, Sigma), 100 U/ml superoxide dismutase (SOD, Sigma), 50 μM EUK-8 (Sigma), 500 μM apocynin (Sigma) and/or 20 μM ML171 (Tocris) for 2 h, or transfected with NOX1 (20 nM) or Trx1 siRNA (20 nM) for 24 h, followed by the treatment with cambogin (10 μM) for 24 h. After treatment, 3-(4, 5-dimethylthiazol-2-yl) 2, 5-diphenyltetrazolium bromide (MTT, Sigma) solution was added to the cells and the mixture was further incubated for 4 h at 37°C. After removing the medium 100 μl DMSO was added and the absorbance was measured at 570 nm, results were normalized as the percentage of control as readout of cell viability.

### Mitochondrial transmembrane potential (Δ*Ψ*_m_) assessment

The electrical potential difference across inner mitochondrial membrane (Δ*Ψ*_m_) was monitored using the Δ*Ψ*_m_ specific fluorescent probe JC-1 (Santa Cruz), a sensitive fluorescent dye. The JC-1 dye changes color as the membrane potential increases. At higher membrane potentials, JC-1 forms aggregates, resulting in changes of the fluorescence emission color from green to red. After treatment, cells were incubated with JC-1 (10 μM) for 20 min at 37°C (protected from light). After incubation, stained cells were observed under a fluorescent microscope and then were assessed by microplate fluorescence reader. All experiments were carried out three times independently and five images per sample were selected randomly and blindly.

### Mitochondrial network imaging acquisition and length measurements

The mitochondrial network was analyzed by staining with the mitochondria-targeting dye MitoTracker Red CMXRos (Invitrogen) as previously described [57] with minor modifications. Briefly, the cells were washed with Hank's balanced salt solution (HBSS), and stained with MitoTracker Red CMXRos (20 nM) in HBSS for 1 h at 37°C (protected from light). For confocal images, samples were obtained and acquired using Leica TCS SP8 equipped with digital inverted microscope at ×1000 magnification. Images were enlarged and analyzed for mitochondrial length using LAS.F 3.3 software in a blind manner. Five images per sample were taken randomly and blindly for three independent experiments.

### Measurement of ROS

After treatment, cells were stained with dihydroethidium (DHE, Sigma), 2′, 7′-dichlorodihydrofluorescein diacetate (DCFH-DA, Sigma), or MitoSOX™ (Invitrogen), to detect O_2_^.-^production, H_2_O_2_ production, or mitochondria O_2_^.-^ production, respectively. After incubation for 30 min at 37°C (in the absence of light), stained cells were observed under a fluorescent microscope (Olympus, Japan) and assessed by microplate fluorescence reader.

### Immunoprecipitation

After treatment, cells were lysed in 0.3% (w/v) CHAPS buffer (1 M HEPES pH7.5, 120 mM NaCl, 1 mM EDTA pH8, 10 mM sodium pyrophosphate, 10 mM β-glycerolphosphate, 50 mM NaF, 0.5 mM sodium orthovanadate, 0.3% CHAPS, 1 mM benzamidine-HCl, 0.2 mM phenylmethylsulfonyl fluoride, 1 μg/ml each of leupeptin and pepstatin). Protein lysates were spun for 10 min at 16,000*g*. The supernatants were kept, and total protein concentrations were determined using the Bradford assay. Lysates containing 1.2 mg of total protein were incubated with anti-Trx1 antibody or anti-p22^phox^ antibody for 16 h at 4°C with rotation, followed by the incubation with protein A/G PLUS agarose beads for a further period of 2 h at 4°C with rotation. Beads were washed three times with 0.3% (w/v) CHAPS buffer, and the immunocomplexes were released by heating in a 2× Laemmli sample buffer and analysed by western blotting.

### SDS-PAGE and Western blotting

After treatment, cells were lysed in RIPA buffer (Cat. #: 9806, Cell Signaling Technology) containing 1 mM PMSF and protease inhibitor cocktail. Lysates were spun at 10,000*g* for 10 min and the supernatants were kept at −20°C until use. SDS-PAGE and Western blotting were performed as previously described [21]. Please see Table 1 for a complete list of antibodies used in this study.

**Table 1:** List of primary antibodies used in this study

*Primary antibody*	*Obtained from*	*Cat. No.*	*WB/IHC Dilution*	*Application*
p-p47^phox^ S304	Bioworld	BS4600	1:500	WB
p22^phox^	SCB	sc-130551	1:200/1:50	WB/IF
NOX1	abcam	ab55831	1:1000/1:500	WB/IF
Trx1	SCB	sc-13526	1:200/1:10	WB/IP
p-JNK/SAPK Thr183 and Tyr185	CST	4668	1:1000	WB
JNK/SAPK	CST	9258	1:1000	WB
p-ASK1 Thr845	SCB	sc-109911	1:200	WB
ASK1	CST	8662	1:1000	WB
GAPDH	CST	2118	1:1000	WB
caveolin-1	CST	3238	1:1000	WB

Dilution ratios given in the third lane are for WB, IF or IP. Abbreviations: CST: Cell Signalling Technology, USA; SCB: Santa Cruz Biotechnology, USA; Bioworld: Bioworld Technology, USA; WB: western blotting; IF: immunofluorescence; IP: Immunoprecipitation.

### Immunofluorescence assay

After treatment, cells were fixed with 4% paraformaldehyde (PFA) for 30 min at room temperature. Fixed cells were blocked with 10% BSA in PBS for 1 h. The cells or tumor tissue sections (4 μm) were incubated with the primary antibodies against NOX1 and p22^phox^ overnight at 4°C followed by Alexa-Fluor 488-conjugated goat anti-rabbit IgG antibody and Alexa-Fluor 594-conjugated donkey anti-mouse IgG antibody for 2 h at room temperature. 4′6-diamidino-2-phenylindole (DAPI, Invitrogen) staining was then used to stain nuclei. For confocal images, samples were obtained randomly and acquired using Leica TCS SP8 equipped with digital inverted microscope at ×630 magnification. Images were enlarged and analysed for PCC using LAS.F 3.3 software in a blind manner.

### NOX activity assay

Cambogin treated MCF-7 cells were harvested and NOX activity was determined as previously described with minor modifications [58]. Briefly, cells were harvested and lysed in lysis buffer (20 mM potassium phosphate, pH7.0, 1 mM EDTA, 10 μg/ml aprotinin, 0.5 μg/ml leupeptin, 0.7 μg/ml pepstatin, and 0.5 mM PMSF) after treatment. Protein lysates were spun for 20 min at 20,000*g*. The precipitates were washed with lysis buffer and then dissolved in 200 μl oxidase assay buffer (50 mM potassium phosphate buffer, pH 7.0, 1 mM EDTA, 150 mM sucrose, 10 μg/ml aprotinin, 0.5 μg/ml leupeptin, 0.7 μg/ml pepstain, and 0.5 mM PMSF). Membrane samples (50 μg of total protein) were incubated with 5 μM lucigenin, 100 μM NADPH, and NOX activity was measured immediately by measuring the luminescence produced by each sample at 37°C. The emission of luminescence was recorded during 30 s intervals for 3 min. Results were blanked against background levels of luminescence. NOX activity is presented as the average luminescent counts per min (CPMs) per mg of protein.

### *In vivo* animal study

BALB/c female nude mice (7 weeks old) were purchased from the Experimental Animal Center of Chinese Academy of Science (Shanghai, China). The experimental procedures were approved by the Shanghai University of Traditional Chinese Medicine Committee on the Use of Live Animals for Teaching and Research, and were carried out in accordance with the Guide for the Care and Use of Laboratory Animals, published by the National Institutes of Health (publication No. SCXX(HU) 2007-0005). MCF-7 breast cancer xenografts were performed as described [59]. Nude mice (n=6 each group) were given subcutaneous injections of MCF-7 cells (3×10^6^ cells per mouse) into the mammary fat site. Before inoculation, 17β-estradiol (E2) pellets (1.7 mg, 60-day release, produce 0.3-0.4 nmol/L E2 (blood levels); Innovative Research of America, Sarasota, FL) were implanted subcutaneously followed by the sealing of the incision with tissue adhesive Vetbond. After tumors had established (~50 mm^3^), nude mice were divided randomly into two groups. Solvent control (0.5% DMSO and 0.5% Tween-80 in normal saline) and cambogin (10 mg/kg in solvent control) were given via intraperitoneal injections every other day. After transplantation, the body weight and tumor sizes of all mice were recorded every three days. Tumor size was determined by Vernier caliper measurements and calculated as [(length×width^2^)/2]. After 35 days of treatment, mice were sacrificed and their tumors were removed, weighed, photographed, and fixed in 4% PFA for immunofluorescence assay.

### SYBR Green assay

Where indicated, cells were pretreated with 1000 U/ml CAT, 100 U/ml SOD, 50 μM EUK-8, 500 μM apocynin and/or 20 μM ML171for 2 h, or transfected with NOX1 (20 nM) or Trx1 siRNA (20 nM) for 24 h, followed by the treatment with cambogin (10 μM) for 24 h. After medium was removed, 100 μl of SYBR Green (1:10000) in lysis buffer were added and the fluorescence intensity units (FIU) were measured at 485 nm (excitation wavelength) and 530 nm (emission wavelength).

### Measurement of NO production

NO production was measured by using a NO assay kit (Cat. #: S0023, Beyotime Institute of Bitotechnology) as previously described with minor modifications [60]. Briefly, cambogin treated MCF-7 cells were harvested and lysed in lysis buffer for NO assay (#S3090, Beyotime Institute of Bitotechnology). The production of NO was determined by measuring the levels of nitrite according to the Griess method. The absorbance was measured at 540 nm, and the production of NO from the samples was calculated using increasing concentrations of sodium nitrite as a reference standard curve.

### Measurement of H_2_O_2_ production

The production of H2O2 was measured by using a H_2_O_2_ assay kit (Cat. #: S0038, Beyotime Institute of Bitotechnology) as previously described with minor modifications [61]. Briefly, cambogin treated MCF-7 cells were harvested and lysed in lysis buffer supplied by the kit. The absorbance was measured at 560 nm. Concentrations of hydrogen peroxide from the samples were calculated by interpolation from the standard curves established using reagents provided by the kit.

### Statistical analysis

Data are presented as means±SEM. Statistical analysis was performed using one- or two-way ANOVA for multiple comparisons or Student's unpaired *t*-test for single comparisons. *P* values less than 0.05 were considered to indicate statistically significant differences.

## SUPPLEMENTARY MATERIALS FIGURES


