# Supplementary material for: Cambogin exerts anti-proliferative and pro-apoptotic effects on breast adenocarcinoma through the induction of NADPH oxidase 1 and the alteration of mitochondrial morphology and dynamics
Source: Oncotarget. 2016 Jul 13;7(31):50596–611. doi: 10.18632/oncotarget.10585 (PMC5226606; doi:10.18632/oncotarget.10585)
Supplement: Supplementary file 1 [file oncotarget-07-50596-s001.pdf]

Cambogin exerts anti-proliferative and pro-apoptotic effects on breast adenocarcinoma through the induction of NADPH oxidase 1 and the alteration of mitochondrial morphology and dynamics

SUPPLEMENTARY FIGURES

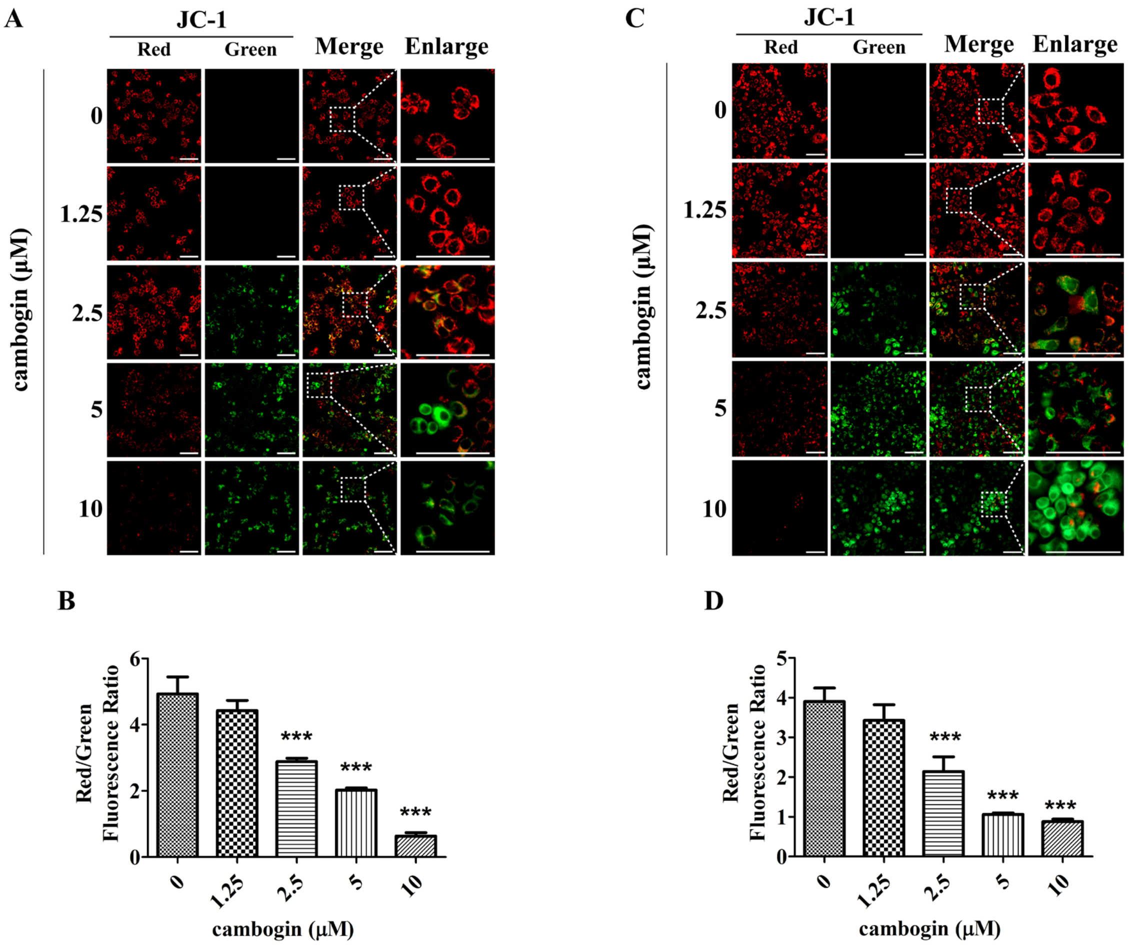

Supplementary Figure S1: Cambogin induces the depolarization of mitochondrial transmembrane potential in breast cancer cells. A, B. MDA-MB-468 cells were exposed to cambogin (0-10 μM) for 4 h. The percentage of cells with a reduction in $\Delta\Psi m$ was measured using the fluorescent dye JC-1 (10 μM). After incubation, stained cells were observed under an inverted fluorescent microscope (A) and were measured by microplate fluorescence reader (B). JC-1 dye changes color as the membrane potential increases. At higher membrane potentials, JC-1 forms aggregates, which changes the fluorescence emission color from green to red. Scale bar=100 μm. **C, D.** SK-BR-3 cells were exposed to cambogin (0-10 μM) for 4 h. The percentage of cells with a reduction in $\Delta\Psi m$ was measured using the fluorescent dye JC-1 (10 μM). After incubation, stained cells were observed under an inverted fluorescent microscope (C) and were measured by microplate fluorescence reader (D). JC-1 dye changes color as the membrane potential increases. At higher membrane potentials, JC-1 forms aggregates, which changes the fluorescence emission color from green to red. Scale bar=100 μm. Data are shown as means±SEM; $^{***}P<0.001$ compared with control. *n*=3.

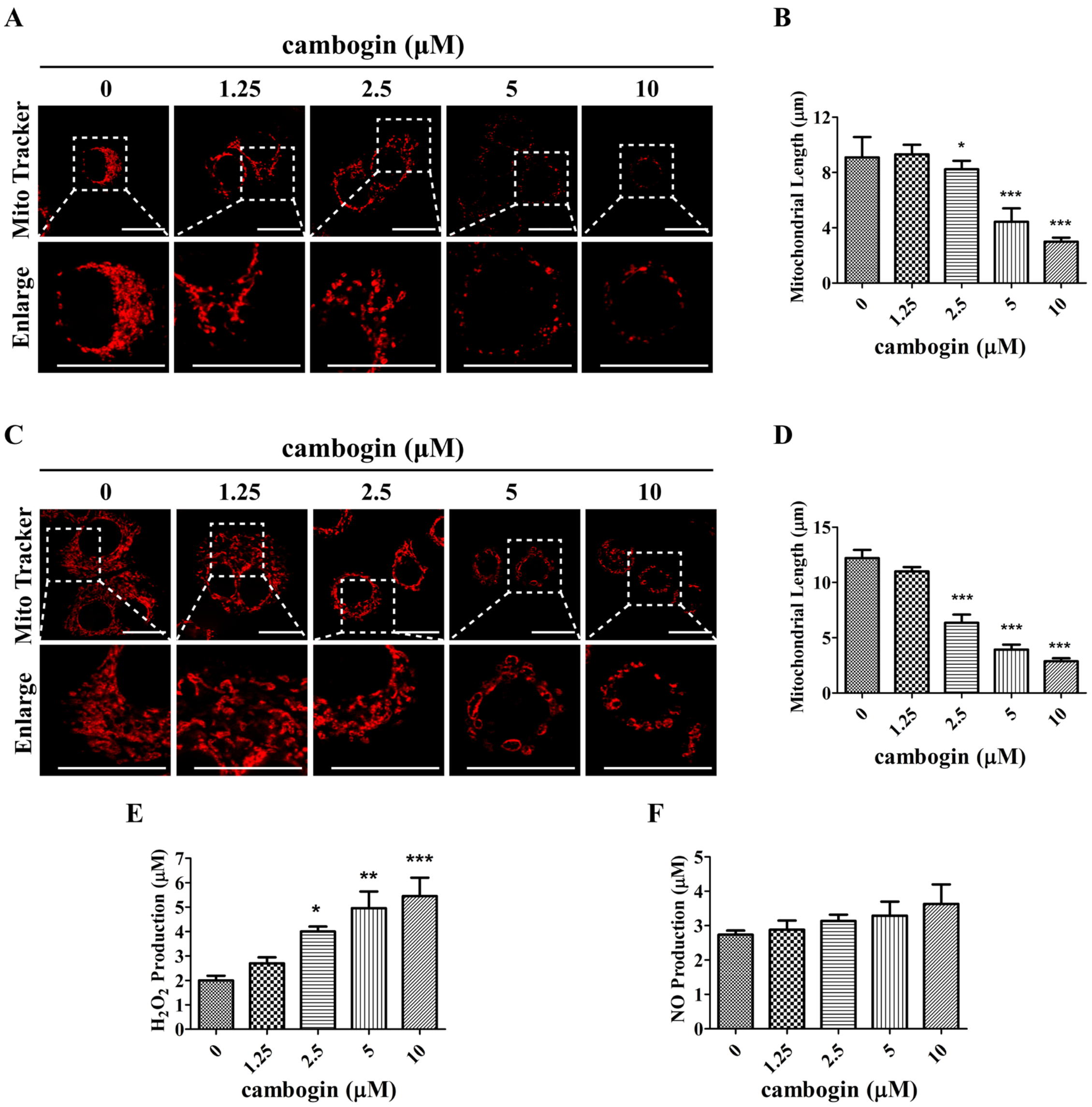

Supplementary Figure S2: Cambogin modulates mitochondrial network in breast cancer cells. **A.** MDA-MB-468 cells were treated with cambogin (0-10 μM) for 24 h. After treatment, the cells were washed, stained with MitoTracker Red for 1 h, washed again, and analyzed for mitochondrial network under confocal microscopy (×1000). Scale bar=20 μm. **B.** Statistical analyses of the average mitochondrial length for experiment A. **C.** SK-BR-3 cells were treated with cambogin (0-10 μM) for 24 h. After treatment, the cells were washed, stained with MitoTracker Red for 1 h, washed again, and analyzed for mitochondrial network under confocal microscopy (×1000). Scale bar=20 μm. **D.** Statistical analyses of the average mitochondrial length for experiment C. **E.** MCF-7 cells were treated with cambogin (0-10 μM) for 2 h. The production of H_2O_2 was measured using H_2O_2 kit as Material and Methods described. **F.** MCF-7 cells were treated with cambogin (0-10 μM) for 2 h. The cellular production of NO was determined with Griess reagent assay. Data are shown as means±SEM; $^{*}P<0.05$, $^{**}P<0.01$, $^{***}P<0.001$ compared with control. *n*=3.

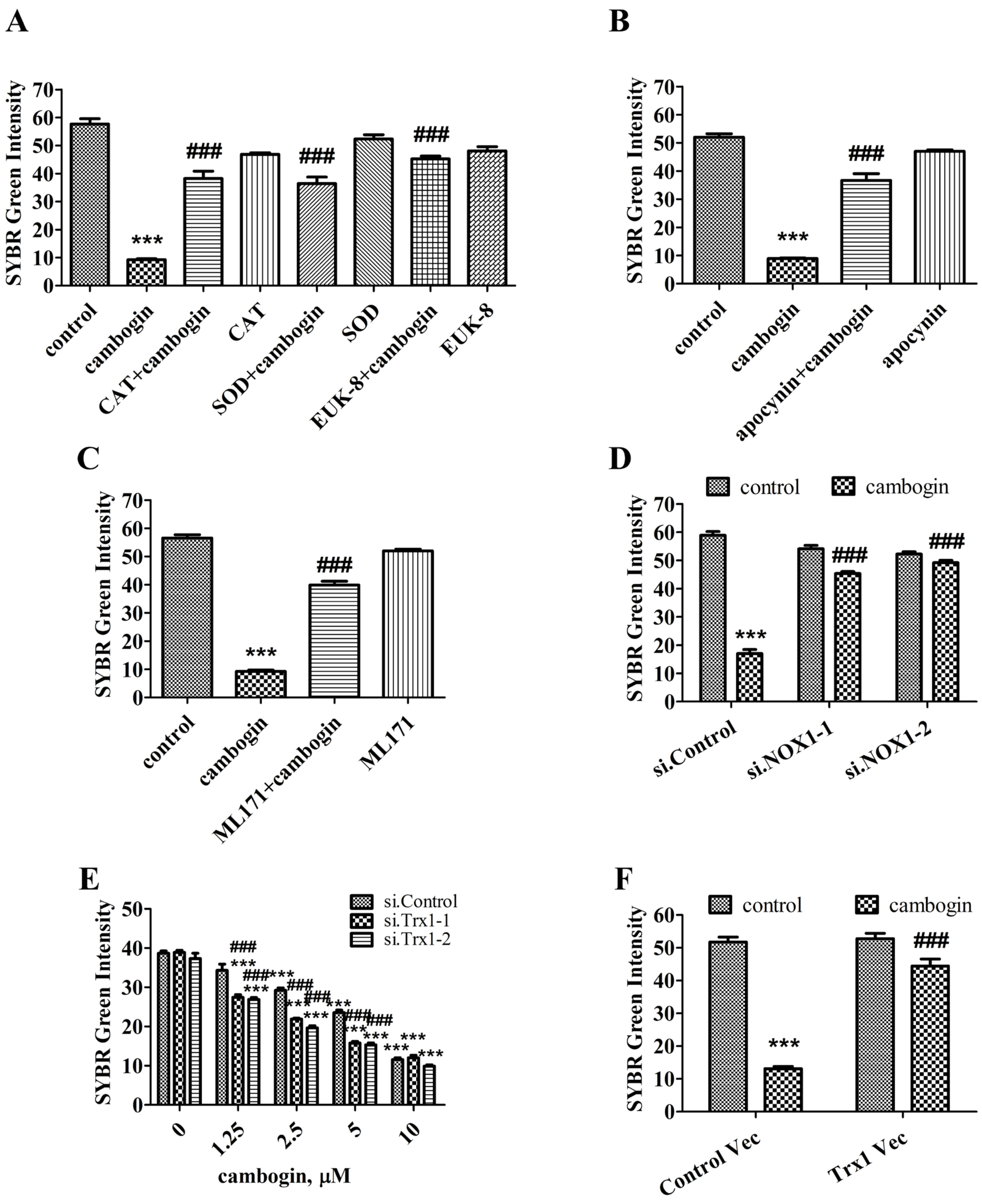

Supplementary Figure S3: The reduction in cell viability evoked by cambogin can be rescued by antioxidants, knocking down Nox1, or over-expressing Trx1. A. MCF-7 cells were treated with cambogin for 24 h after pretreatment with CAT (1000 U/ml), SOD (100 U/ml), and EUK-8 (50 μM) for 2 h. **B.** MCF-7 cells were treated with cambogin for 24 h after pretreatment with apocynin (500 μM) for 2 h. **C.** MCF-7 cells were treated with cambogin for 24 h after pretreatment with ML171 (20 μM) for 2 h. **D.** MCF-7 cells were treated with cambogin for 24 h after transiently transfected with two independent NOX1 siRNAs (si.NOX1-1 and si.NOX1-2) or control scrambled siRNA (si.Control). **E.** MCF-7 cells were treated with cambogin for 24 h after transiently transfected with two independent Trx1 siRNAs (si.Trx1-1 and si. Trx1-2) or control scrambled siRNA (si.Control). **F.** MCF-7 cells were treated with cambogin for 24 h after transiently transfected with Trx1 plasmid (Trx1 Vec) or control plasmid (Control Vec). Cell viability was measured by SYBR Green assay. Data are shown as means±SEM; $^{***}P<0.001$ compared with control; $^{\#\#\#}P<0.001$ compared with cambogin-treated cells. $n=3$.

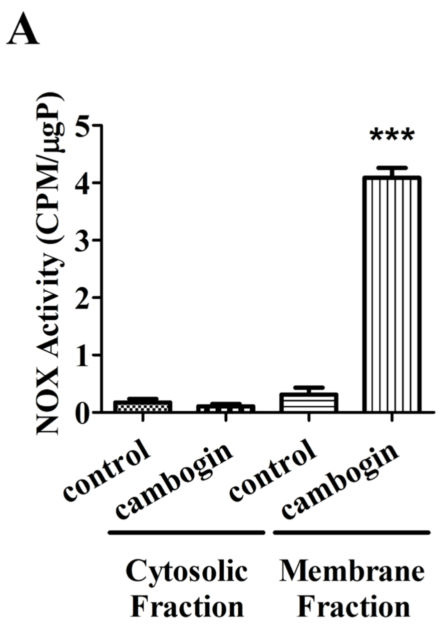

Supplementary Figure S4: Cambogin evokes an increase in NOX activity in the membrane fractions of breast cancer cells. A, B. Breast cancer cells were incubated in the absence or presence of cambogin (10 μM) for 2 h in MDA-MB-468 (A) and SK-BR-3 (B) NOX activity was measured and the enzyme activity is expressed as relative light units (RLU). Data are shown as means±SEM; $^{***}P<0.001$ compared with control. *n*=3.
